# Gut microbiome dysbiosis and correlation with blood biomarkers in active-tuberculosis in endemic setting

**DOI:** 10.1371/journal.pone.0245534

**Published:** 2021-01-22

**Authors:** Aasia Khaliq, Resmi Ravindran, Samia Afzal, Prasant Kumar Jena, Muhammad Waheed Akhtar, Atiqa Ambreen, Yu-Jui Yvonne Wan, Kauser Abdulla Malik, Muhammad Irfan, Imran H. Khan

**Affiliations:** 1 School of Biological Sciences, University of the Punjab, Lahore, Pakistan; 2 Department of Pathology and Laboratory Medicine, University of California, Davis, California, United States of America; 3 Kauser Abdulla Malik School of Life Sciences, Forman Christian College, Lahore, Pakistan; 4 Department of Microbiology, Gulab Devi Hospital, Lahore, Pakistan; Rutgers Biomedical and Health Sciences, UNITED STATES

## Abstract

Tuberculosis (TB) is the largest infectious disease with 10 million new active-TB patients and1.7 million deaths per year. Active-TB is an inflammatory disease and is increasingly viewed as an imbalance of immune responses to *M*. *tb*. infection. The mechanisms of a switch from latent infection to active disease is not well worked out but a shift in the immune responses is thought to be responsible. Increasingly, the role of gut microbiota has been described as a major influencer of the immune system. And because the gut is the largest immune organ, we aimed to analyze the gut microbiome in active-TB patients in a TB-endemic country, Pakistan. The study revealed that *Ruminococcacea*, *Enetrobactericeae*, *Erysipelotrichaceae*, *Bifidobacterium*, etc. were the major genera associated with active-TB, also associated with chronic inflammatory disease. Plasma antibody profiles against several *M*. *tb*. antigens, as specific biomarkers for active-TB, correlated closely with the patient gut microbial profiles. Besides, *bcoA* gene copy number, indicative of the level of butyrate production by the gut microbiome was five-fold lower in TB patients compared to healthy individuals. These findings suggest that gut health in TB patients is compromised, with implications for disease morbidity (e.g., severe weight loss) as well as immune impairment.

## Introduction

The etiologic agent of tuberculosis (TB), *Mycobacterium tuberculosis* (*M*. *tb*.), latently infects a third of the world’s population (approximately two billion) and leads to an estimated 10.4 million new cases of active disease (active TB) every year [1]. Active TB is responsible for 1.7 million deaths each year, making it the biggest infectious disease killer, ahead of HIV-AIDS (1.0 million deaths) and malaria (0.44 million deaths) [2]. What is even more alarming about TB is while deaths due to the other two leading infectious diseases (AIDS and malaria) are trending down TB deaths are steadily trending up with no clear signs of reversal on the horizon [1,2].

The shift from latent *M*. *tb*. infection to active disease occurs in about 10% of the infected individuals, sometime during their lifetime. It is thought to be a result of alterations in the immune balance, however, the trigger(s) of this shift remains poorly understood [3]. The pivotal role played by the immune system in the progression to active TB suggests the disease is primarily an immune pathology, but the mechanism(s) are not clearly defined [3]. Accordingly, understanding of the immune responses in TB patients is of crucial importance. The involvement of the cells of the host immune system begins in the earliest stages of infection when *M*.*tb*. infects the lung and continues throughout infection and disease. The bacteria are taken up by alveolar macrophages and dendritic cells (DCs), triggering inflammatory responses [3,4]. This is followed by the recruitment of monocytes and polymorphonuclear neutrophils to the site of infection; these cells express diverse antimicrobial effector molecules to activate macrophages and escalate the inflammatory process [5]. Antigen-presenting DCs activate T lymphocytes in the lymph node, which then migrate to the site of infection and proliferate, leading to the formation of granulomas, a hallmark of *M*.*tb*. infection. Areas of high lymphoid cell activity, arranged in tertiary lymphoid structures develop around lung granulomas and have been suggested to mimic lymphoid organs in their function [6]. To define pathologic mechanisms of TB, the roles of cytokines and chemokines have been extensively studied as their dynamics play a key role in the disease outcome [6–8]. Proinflammatory cytokines help in the control of *M*. *tb*. infection, but they also play a crucial role during the chronic (latent) infection stage, dictating the pathogenesis of the disease [9]. Activated T cells regulate the flow of inflammatory events by secreting IFN-γ and IL-2, which activate alveolar macrophages to produce a variety of substances involved in growth inhibition and killing of mycobacteria [10]. In addition to the cell-mediated immunity by T cells (involving cytokines/chemokines) and mononuclear cells, humoral immune responses in TB immunity are also critical [11,12]. Importantly, these studies have demonstrated the importance of the co-operative roles of innate, humoral and cellular immune responses. B cells, and their influence on the TB immunity through the production of antibodies, have come to be considered key players in the host defenses [11,12]. In addition to the production of antibodies crucial for TB immunity, B cells contribute to immune responses through antigen presentation and cytokine production [12].

The impact of immune balance (or imbalance) on the progression of active disease (TB) has been substantially documented [13]. Considering the gut is the largest immune organ, and the gut microbiota plays a key role in the modulation of immune responses, it is important to analyze the gut microbiota [14]. Gut microbiota has been shown to support the induction of vaccine responses, anti-pathogen interferon responses, and maintenance of memory cell populations [15–17]. Although studies on animal models have contributed substantially to the understanding of immune responses in active TB, no models, including nonhuman primate model, accurately yield the information as that obtained in human subjects [18]. Several studies investigating perturbations in the gut microbiome during *M*.*tb*. infection and the effect of anti-TB drug therapy on the gut microbiome composition are currently emerging in human [19–23].

The possible relationship between gut microbiome dysbiosis and blood immune-biomarkers in active-tuberculosis in the endemic setting has not been evaluated. We have prviously shown that anti-*M*.*tb*. antibodies are specific diagnostic biomarkers for TB as opposed to cytokines which are general immune reponse biomarkers [24–26]. To better understand the relationship of immune responses and gut microbiota we analyzed plasma antibodies against several *M*. *tb*. antigens. In this study, we present data showing perturbations of the gut microbiota in human patients in a TB endemic country. The patients and healthy controls were from the same geographic area with the assumption they were all exposed to similar environmental conditions, including pathogens (viruses and bacteria). Our results show gut microbes, at the genus level, inhabiting TB patients are distinct from those in healthy individuals. In addition, we have found strong correlation between antibodies that are high in active TB patients with microbiota that are enriched in TB. These results suggest the gut microbiota dysbiosis observed in TB patients may be relevant to TB pathogenesis.

## Material and methods

### Patient recruitment and sample collection

Patient recruitment was done from the Outdoor patient department (OPD) of Gulab Devi Hospital (GDH), Lahore, Pakistan, from January 2017 to June 2017. GDH is a tertiary care hospital located in Lahore (capital of the Punjab province, and country’s second largest city) and one of the largest chest specialty hospitals. Patients throughout the province Punjab are referred here for diagnosis and treatment, so the samples collected from here can be considered as representative of a large population. GDH follows the World Health Organization (WHO) guidelines for the diagnosis and treatment of pulmonary TB patients as previously described (19). Briefly, patients are screened by chest x-ray (CXR) and acid-fast bacilli sputum smear microscopy (AFB microscopy) for two consecutive samples (spot and early morning). Patients positive for AFB smear for at least one sample are considered positive (AFB+), and their anti-TB treatment (ATT) is immediately initiated. For AFB- cases, the patient is prescribed broad spectrum antibiotics (Amoxicillin 500mg and Co-trimoxazole combined with Trimethoprim, 100mg) for 2 weeks, followed by another round of AFB microscopy and CXR. If the CXR is suggestive and the clinical symptoms consistent with pulmonary TB persist, the patient is considered a AFB- pulmonary TB patient, and ATT is initiated. In this study, sputum smear-positive by AFB-microscopy (Category-1), who did not have any previous history of TB infection or ATT were included in the study. TB patients were only recruited when their diagnosis was finalized by the physician and they were registered in GDH for 6 months ATT course. All patients were screened for HIV. HIV positive active-TB patients were excluded from the study. Healthy individuals (staff and students of Forman Christian College (a chartered university), FCCU, Lahore) who did not have close contact with active TB patients at present or in the previous at least one year and had not taken any broad-spectrum antibiotics in the previous six months and with no major illness were included in the study during the same period. A total of 82 subjects (TB patients n = 42, Healthy n = 40) of both genders were included in this study. All the subjects were of Pakistani origin. Clinical history information including fever, cough, hemoptysis, night sweats, loss of appetite, weight loss, previous history of TB or ATT and co-morbidity of the diseases like diabetes, Chronic obstructive Pulmonary Disease (COPD) and asthma was taken. Demographic variables like age, gender, BCG vaccination, smoking, occupation from each participant were recorded on the questionnaire. Sample processing was done in FCCU, Lahore,. and analysis was done at the School of Biological Sciences, University of Punjab (SBS, PU), and the University of California, Davis, USA.

### Ethical approval

The study was approved by the Ethical Review Committee of FCCU (ERC- 23–2016). To all study participants (TB patients & healthy individuals) the objectives of the study were explained in their native language. Written consent was obtained from all those participants who agreed to participate in the study. All the TB patients who didn’t give consent were not included in the study but this decline of consent did not affect their treatment regimen. After the written consent, blood, stool and sputum samples were obtained from TB patients while from healthy individuals, blood and stool samples were taken according to the Standard Operating Procedures (SOPs) approved by the ethical review board of the institution.

### Sample processing

#### Sputum samples

Two separate sputum (spot and early morning) samples were taken from each TB patient. Both samples were processed for AFB-microscopy (Ziehl-Neelsen (ZN) staining) in Microbiology Laboratory at GDH Lahore. For culturing, sputum samples were processed for liquefaction and decontamination by the NALC (N-acetyl L-cysteine) method followed by culture on solid LJ (Löwenstein–Jensen) media and liquid MGIT-960 (Mycobacteria Growth Indicator Tube-960) [27].

#### Blood samples

Blood samples (5 ml) were collected into a Vacutainer tube (EDTA, catalog # 367899; BD, Franklin Lakes, NJ) via venipuncture. Plasma was separated by centrifugation at1500 x *g* for 10 min from blood samples for TB patients and healthy controls and frozen in aliquots at −80°C until further use.

#### Stool samples

Fresh stool samples (morning samples) were taken in wide-mouth containers with covers from both TB patients and healthy controls.

### HIV screening

A rapid HIV testing kit (Advance Quality Rapid Anti-HIV (1 & 2) Test Card (whole blood/serum/plasma) by Intec Products Inc. Xiamen, China; Catalog Number: ITP02002) was used for HIV testing in TB patients and healthy controls. All TB patients and healthy controls included in this study were HIV negative by this method. It is important to note that Pakistan is among the lowest HIV prevalence (general population) countries worldwide [26].

### DNA extraction from stool samples

DNA extraction from stool samples was performed using FavorPrep^TM^ Stool (Catalog # FASTI 001–1, FAVORGEN Biotech Corp. Taiwan) per manufacturer’s instructions within two hours of collection [28]. Briefly, stool sample was added in a tube with beads along with proteinase K (10 mg/ ml) and SDE1 (Sequential detergent Extraction) buffer and vortexed for 5 minutes followed by incubation at 60°C for 20 min. After homogenization, samples were incubated at 95°C for 5 min followed by the addition of SDE2 buffer, vortex, incubated for 5min and then centrifuged at 18,000 x *g* for 5 min. To the supernatant SDE3 buffer was added, vortexed and incubated for 2 min followed by centrifugation at 18,000 x *g* for 2 min. In 250 μL of supernatant, 1 μL of RNase A was added and incubated for 2 min. An equal volume of ethanol (96~100%) and SDE4 buffer were added, pulse-vortexed and then transferred to SED column. The column was centrifuged at 18,000 x *g* for 1 min, 750ul of wash buffer was added, and centrifuged twice at 18,000 x *g* for 1 and 3 min respectively. To the SDE column, 75μl of preheated elution buffer was added, incubated at room temperature for 2 min and then centrifuged at 18,000 x *g* for 1 min to elute DNA [29].

### DNA quantification

DNA extracted from stool samples was quantified by Nano Drop (NanoDropTechnologies, Thermo Scientific, Wilmington, MA).

### Analysis of 16 sRNA and IS 6110 PCR

Universal primers were used to amplify a large fragment of the 16S rRNA gene for prokaryotes. The primer sequence was:

Forward primer P1 (5'-CGGGATCCAGAGTTTGATCCTGGTCAGAACGAACGCT-3'

Reverse primer P6 (5'-CGGGATCCTACGGCTACCTTGTTACGACTTCACCCC-3'

For IS6110 PCR following primer sequences were used for amplification of 200 bp. region:

IS Forward 5’ CCTGTCCGGGACCACCCGCGGCAA 3’

IS Reverse 5’ GGATCCTGCGACGTAGGCGTCGG 3'

DNA sample of H37Rv (obtained from National Reference lab, Islamabad, Pakistan) was used as a positive control for IS6110 PCR. Amplified PCR products were visualized on an agarose gel. The stool DNA samples which were positive for IS6110 PCR confirmed the presence of *M*.*tb*. Complex (MTC). These samples were further subjected to PCR with *rpoB* primers to amplify the 80 bp. hotspot region of *rpoB* gene which confirmed the presence of *M*.*tb*. in the samples. The primer sequence for *rpoB* is as follows:

*rpoB* Forward: CGATCAAGGAGTTCTTCGGCACC

*rpoB* Reverse: AGGGGTTTCGATCGGGCACATCC

The PCR reaction mix was made by using master mix (Qiagen Hotstar Taq Plus Master Mix Kit Cat # 203643). The reaction mixture was made for 25 ul (7ul of master mix, 2.5ul of each forward and reverse primer, 3ul DNA(30ng/ul) and 10ul water). The optimized PCR conditions used were: 94°C for 5min (initial denaturation), 94°C, 30 sec (denaturation); 55°C, 30sec (annealing); 72°C, 30sec (extension) (35 cycles), 72°C, for 10 min. PCR products were analyzed on 2% agarose gel. The stool samples positive for 16S rRNA, IS6110 and *rpoB* PCR were sent for sequencing.

### Analysis of *bcoA* gene copy number by qPCR

PCR amplification from stool DNA was performed using *bcoA* primers with the following sequence:

Forward: GCIGAICATTTCACITGGAAYWSITGGCAYATG;

Reverse: CCTGCCTTTGCAATRTCIACRAANGC based on the published sequences [30,31].

Synthetic DNA fragments of *bcoA* gene were used as a standard. Known concentrations of the standard template DNA fragments were serially diluted by ten-fold dilutions and bacterial *bcoA* gene copy number in the stool DNA was extrapolated from the known amounts of the synthetic *bcoA* gene fragments, as previously described [16,32].

### 16S rRNA gene sequencing to study profiles of microbiota

16S library preparation was performed based on Nextera XT DNA Library Preparation Kit (Illumina Inc., Hayward, CA, USA). Amplification of variable region 4 (V4 region) of 16S rRNA, barcoding, and pooling of amplicons were performed according to the manufacturer’s instructions [33]. After each PCR amplification, DNA concentration and fragment size were measured on a Qubit fluorometer (Invitrogen, Carlsbad, CA, USA) and agarose gel. DNA purification was performed using the QIAquick PCR Purification Kit (Qiagen, Valencia, CA, USA). A pooled library was prepared and checked for quality and purity using Agilent Bioanalyzer chip with Agilent High Sensitivity DNA kit (Agilent Technologies, Waldbronn, Germany) [33]. Illumina sequencing of the pooled library of 16S rRNA gene amplicons was performed as previously described [34,35]. The V4 region of 16S rRNA gene was sequenced using Illumina MiSeq. Sequence reads were analyzed using QIIME software version 1.9.1 [36].

Sequence reads down to the genus level were targeted. However, where sequence information did not reach the genus level, the microbe was represented by family name with ‘_g’ added to it to signify the unknown genus responsible for the significant difference in the results.

### Microbiome data analysis

#### Bioinformatics and statistical analysis

Sequence reads for 16S rRNA gene amplicons were analyzed using QIIME software (Version 1.9.1). R Statistical Software was used to generate principal component analysis (PCA) plot, heatmap, and spearman correlation [35]. Functional profiles of microbial communities were predicted using PICRUSt (Phylogenetic Investigation of Communities by Reconstruction of Unobserved States) and LEfSe (Linear discriminant analysis Effect Size) was performed to identify differentially enriched pathways [37,38]. All other comparisons were calculated by two-tailed Student’s *t-*test or one-way ANOVA followed by Tukey’s test using Graph Pad Prism 6 software (Graph Pad Software, Inc., La Jolla, CA). *p*-values are adjusted for multiple comparisons using the false discovery rate. *p* < 0.05 was considered statistically significant. From the raw data, phyloseq object was made by using R script. The QIIME package was used to generate Unifrac distance as beta diversity, and Shannon index as alpha diversity.

### Multiplex plasma antibody profiling

Recombinant antigens (11 *M*. *tb*. genes) were expressed in *Escherichia coli* and purified as previously described [39]. Carboxylated microbeads were purchased from Luminex Corp. (Austin, TX). Various antigen preparations were covalently conjugated to the microbeads according to the manufacturer's instructions. Briefly, 2.5 × 10^6^ beads was removed and centrifuged at 21,000 × *g* for 2 min. Beads were resuspended in 80 μL of activation buffer (100 mM monobasic sodium phosphate; pH 6.3) by vortexing and sonication (15 to 30 s). To activate the beads for cross-linking to proteins, 10 μL of 50-mg/ml sulfo-N-hydroxysulfosuccinamide (Pierce, Rockford, IL) was added followed by 10 μL of 50-mg/ml 1-ethyl-3-[3-dimethylaminopropyl] carbodiimide (EDC; Pierce, Rockford, IL) and inubated on a rotary shaker at room temperature for 20 min. Beads were washed twice with 250 μL phosphate-buffered saline (PBS), pH 7.4). To coat them with antigens, pelleted beads were resuspended in the relevant antigen preparation diluted in PBS and incubated by shaking on a rocker for 2 h at room temperature. Beads were washed twice with 250 μL of PBS, resuspended in 250 μL of blocking buffer (1% BSA; 0.1% Tween 20 in PBS, pH 7.4; 0.05% sodium azide), and shaken on a rocker at room temperature for 30 min. After blocking, beads were resuspended in 1 ml of blocking buffer and stored at 4°C in dark.

A multiplex microbead immunoassay based on the xMAP technology platform (Luminex Corp, Austin, TX) was designed to detect the plasma antibodies using uniquely labeled microbeads conjugated with the following recombinant *M*.*tb*. antigens: (Rv3881c, Rv0934 (P38 or PstS1), Rv2031c (HspX), Rv1886c (Ag85b), Rv1860 (MPT32), Rv3874 (CFP10), Rv1926c, Rv1984c (CFP21), Rv3841 (Bfrb1) and Rv2875 (MPT70). In addition, uniquely labeled microbeads were coated with membrane extracts (MEM) from H37RV*M*. *tb*. strains obtained from the TB Resource Center at Colorado State University (Fort Collins, CO) [40]. The assay was performed as previously detailed [41]. In brief, a mixture of microbead sets, one for each of the coated antigens described above, were incubated with the participants’ plasma specimens, which were diluted 1:200 in Prionex (bio-WORLD, Dublin, OH) for 1 hour at room temperature. After incubation, liquid was drained from the bottom of the plate in a vacuum manifold designed to hold 96-well plates (Millipore Corporation, Bedford, MA). The beads were washed two times by adding 100 μL of wash buffer per well and draining under vacuum. For detection of human IgG, phycoerythrin conjugated anti-human IgG was used (Jackson ImmunoResearch, Pennsylvania) at a 1:500 dilution in PBS-tween, and 50 μL was added per well. Beads were mixed as before and incubated at room temperature for 15 min. Following incubation with the secondary antibody, beads were washed two times with wash buffer, resuspended in 100 μL of wash buffer per well, and analyzed in the Luminex-100 instrument.

### Antibody data collection

Data were collected as median fluorescence intensity (MFI). Data for 11 antigens was collected from each sample (in duplicate), resulting in a total of 1,980 data points. Data collected for healthy controls was used to calculate the cut-off values for the 11 *M*.*tb* antigens separately using formula (Cutoff = Mean MFI + (3 × standard deviations). The cut-off values were used to determine antibody-positive samples (at least one antibody) in the data.

### Antibody data and microbiome correlations

To determine the correlations of the eleven *anti-M*.*tb*. antigen antibodies with microbiome genera Spearman’s Correlation was performed with R Statistical Software as previously described [35].

Microbiome and antibody data of same sample were merged by subject and threshold. The microbiome OTU data and antibody data were first combined and filtered to remove low abundance OTUs and antibody MFIs (appearing in less than 50% of samples). The Spearman’s ranked correlation was calculated using package cor.test function of R statistical software. The p-values was then adjusted using p. adjust function before filtering for significant correlations.

## Results

### Human subject characteristics

Characteristics (age, gender, Body mass index (BMI)) of the study participants are shown in Table 1A. The median age of TB patients and healthy individuals was 27 and 40 years, respectively. The difference between median ages is because the volunteers who consented to participate in the study were younger among TB patients than healthy individuals. The gender ratio among the TB patients favored more males than females (ratio: 1.8); we matched the same ratio in healthy controls. Symptoms of TB patients (fever, cough, loss of appetite, hemoptysis, and night sweats), TB-contact history and co-morbidity (e.g. diabetes) are provided in Table 1B. Majority of the TB patients were showing typical symptoms of TB e.g. cough (98%), fever (86%), night sweats (76%), loss of appetite (71%) and weight loss (76%). 24% of the cases were having contact with active TB patients either as household contact or at work place and 24% of the patients have the co-morbidity of diabetes. Diabetes is known to have a major influence on gut microbial dysbiosis [42]. In this study since only 10 TB patients had diabetes, we have not performed any statistical analysis between TB patients with and without diabetes. None of the healthy individuals had diabetes. The detailed clinical history of TB patients and healthy individuals is given in the supplementary data (S1 File).

**Table 1:** A: Human subject characteristics. B: Clinical details of TB patients.

		TB Patients n = 42 (%)	Healthy individuals n = 40 (%)
**Age (median)**		27	40
**Gender (%)**	Male	27 (64)	29(73)
	Female	15 (36)	11(27)
**BMI (%)**	Underweight	16 (38)	16 (40)
	Normal	21 (50)	17 (42)
	Overweight	4 (10)	6 (15)
	Obese	1(2)	1 (3)
		**TB Patients (n = 42)**	
		**Yes (%)**	**No (%)**
**Cough**		40 (98%)	2 (2%)
**Fever**		36 (86)	6 (14)
**Hemoptysis**		8 (19)	34 (81)
**Night sweats**		32 (76)	10 (24)
**Loss of appetite**		30 (71)	12 (29)
**Weight loss**		32 (76)	10 (24)
**History of TB**		1 (2)	41 (98)
**Close Contact of TB**		10 (24)	32 (76)
**Diabetes**		10 (24)	32 (76)

### Microbiota abundance (phyla level)

**Fig 1:**
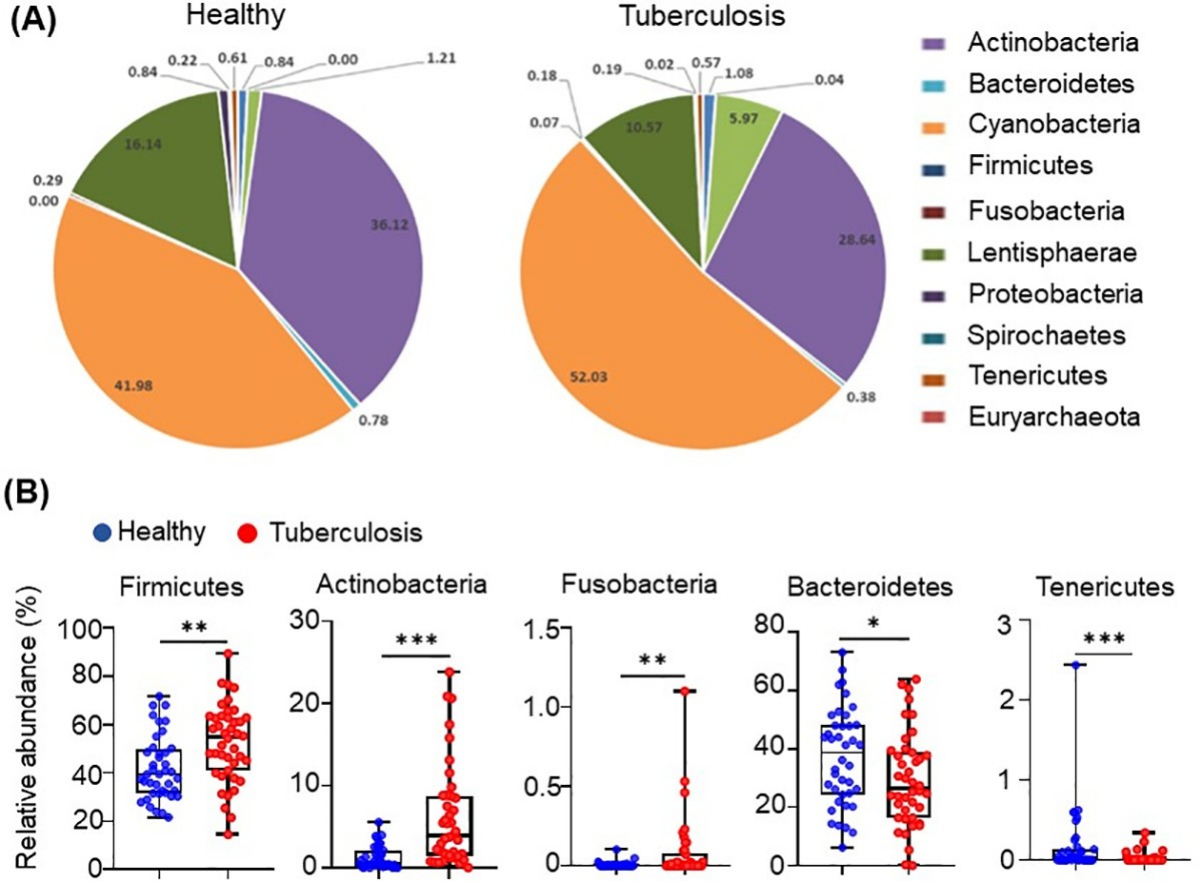
Differences in the abundance of microbiota in active-TB patients and healthy individuals, at the level of phyla. (A) Taxonomic distributions of bacteria from fecal 16S rDNA sequencing data from healthy (n = 40) and tuberculosis cohorts (n = 42). (B) Relative abundances of Firmicutes, Actinobacteria, Fusobacteria, Tenericutes and Bacteroidetes from fecal 16S rDNA sequencing data from healthy (n = 40) and tuberculosis cohorts (n = 42). *p* value statistically significant based on Man-Whitney t-test, *p< 0.05; **p< 0.01 and ***p< 0.001.

Among the 11 most abundant bacterial phyla, three were substantially enriched in TB patients (Fig 1A and 1B). The phyla Fusobacteria and Actinobacteria, which contain many Gram-negative bacteria and opportunistic pathogenic species, were increased 4-fold, (p-value < 0.01) and 2-fold (p-value < 0.001), respectively, in comparison to the healthy group (Fig 1B, Table 2). Firmicutes, the second-largest phylum of gut microbe, was also enriched in TB patients (p-value < 0.01) (Fig 1B, Table 2). On the other hand, the relative abundance of the phylum Bacteroidetes, the largest component of the gut microbe with more than 50% of total abundance, containing many beneficial commensal organisms, and the Phylum Tenericutes, was diminished in TB patients as compared to healthy controls ((p-value < 0.05) and (p-value < 0.001) respectively (Fig 1B; Table 2).

**Table 2:** Log2 Fold change at the phyla level in TB patients and healthy controls.

Taxonomy_Phyla	Fold Change TB vs Healthy (Log2)	p-value
k__Bacteria|p__Fusobacteria	4.884	< 0.01
k__Bacteria|p__Actinobacteria	2.306	< 0.001
k__Bacteria|p__Firmicutes	0.310	< 0.01
k__Bacteria|p__Bacteroidetes	-0.335	< 0.05
k__Bacteria|p__Tenericutes	-3.343	< 0.001

### Diversity of gut microbiota in TB patients

**Fig 2:**
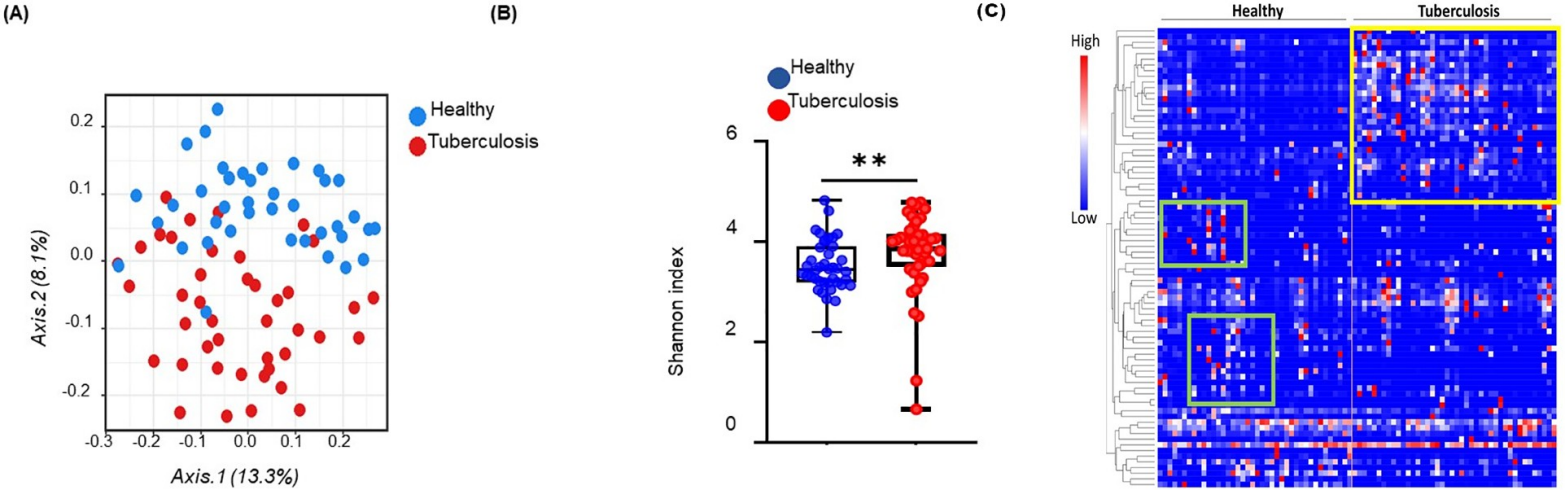
Gut microbiota diversity in tuberculosis patients. (A) Principal coordinates analysis of unweighted UniFrac distance based on 16S rDNA profiling of the gut microbiome from healthy (n = 40) and tuberculosis cohorts (n = 42), *p* value statistically significant based on Bonferroni-corrected parametric t-test, ****p< 0.001. Gut microbiome in active-TB patients (red) and healthy individuals (blue) display separation in the two groups. Each data point represents the entire microbial signature in individual samples. (B) Shannon diversity index of fecal 16S rDNA sequencing data from healthy (n = 40) and tuberculosis cohorts (n = 42). *p* value statistically significant based on Man-Whitney t-test, **p< 0.01. (C) Microbiome profiles (84 genera and families where unknown genus contributed to significant difference in comparison with healthy controls) of microbiota shown in a heat map. Unique clusters of genera in active-TB patients are outlined by a yellow box and those in age-matched healthy individuals by the green boxes. Each small square in the heat map represents a genus. Clusters of bacterial genera in TB patients are well separated from those in healthy individuals.

Scatterplot from PCoA analysis of the operational taxonomic unit (OTUs) shows the differences in microbial community structures among TB patients and healthy controls (Fig 2A). Gut microbiota signatures in TB patients clustered uniquely, separate from those in healthy individuals. PCoA analysis of the whole genome sequences of fecal microbiota based on 16 S sequencing at the genus level is displayed as β-diversity, showing two clusters representing the two groups of subjects (TB patients: red cluster, and healthy: blue cluster). Individual sample data points represent (OTU), where each patient and healthy sample encompasses its own microbial signature (each OTU is a composite of the complete microbial signature of each sample, individually). Positions of OTUs on the plot show the diversity between the individual microbial signatures within each of the two groups. *p* value statistically significant based on Bonferroni-corrected parametric t-test, ****p< 0.001. Fig 2B shows the Shannon diversity index of fecal 16S rDNA sequencing data from healthy and tuberculosis cohorts. p value statistically significant based on Man-Whitney t-test, **p< 0.01.

### Global microbial analysis

Analysis of 84 microbial genera and families (unknown genus contributing to significant difference in comparison with healthy controls) is displayed in a heatmap (Fig 2C). We have performed Supervised-One-Minus Pearson Rank Correlation clustering of the genus level taxa of fecal 16S rDNA sequencing data from healthy and tuberculosis cohorts. The clusters that were boxed and specifically highlighted were identified visually based on the differences between healthy individuals and TB patients. Statistical analysis was not performed on this data set. Clusters of microbiome in TB patients were well separated (with minimal overlap) from those in healthy individuals (TB patients: outlined in the yellow box, and healthy individuals: green boxes). The dominating microbiota in TB patients did not overlap with those in healthy individuals, and vice versa. These results show the global dysbiosis of gut microbiota in active-TB patients.

### Microbiota enriched in active-TB patients

**Fig 3:**
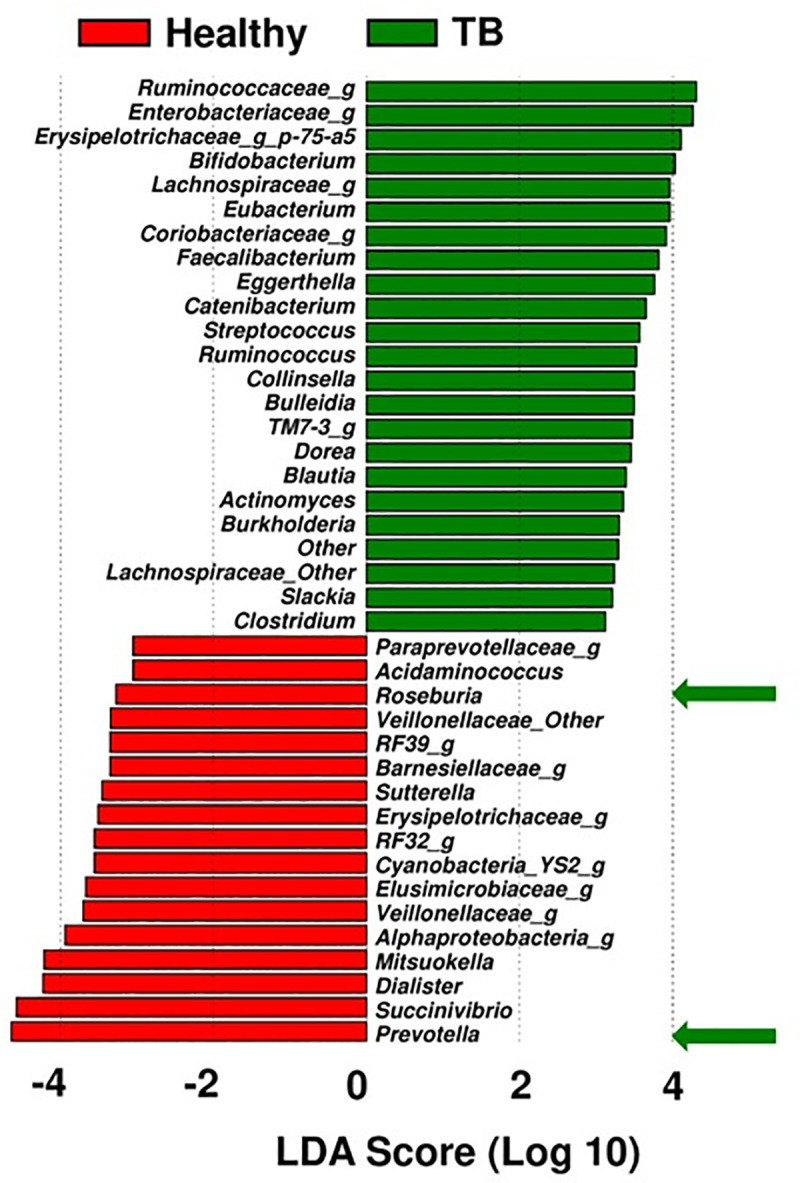
Linear discriminant analysis effect size (LEfSe) of the top 25 significant families and/or genera in active-TB patients and healthy individuals. The linear discriminant analysis scores (LDA) predicate and identify enriched microbes. Bacterial genera or families (unknown genus contributing to significant difference) enriched in active-TB patient group (green bars), in comparison to those in healthy control group (red bars).

Among the top 40 most significantly different microbiota (a mixture of genera and families with unknown genus contributing to significant difference in comparison to healthy controls), 23 were uniquely enriched in active-TB patients, and the other 17 were enriched in healthy individuals (Fig 3). The top enriched families and genera in TB patients, in the descending order of LDA score, are *Ruminococcaceae*, *Enterobacteriaceae*, *Erysipelotrichaceae*, *Bifidobacterium*, *Lachnospiraceae*, *Eubacterium*, *Coriobacteriaceae*, *Faecalibacterium*, *Eggerthella*, *Catenibacterium*, *Streptococcus*, *Ruminococcus*, *Collinsella*, *Bulleidia*, *TM7-3*, *Dorea*, *Blautia*, *Actinomyces*, *Burkholderia*, *Lachnospiraceae other*, *Slackia*, and *Clostridium*. *Prevotella* (phylum: Bacteroidetes) was the most abundant genus in the fecal microbiota in healthy individuals. And the top 17 families and/or genera in healthy individuals were: *Prevotella*, *Succinovibrio*, *Dialister*, *Mitsuokella*, *Alpha Bifidobacterium*, *Veillonellaceae*, *Elusimicrobiaceae*, *Cyanobacteria*, *RF32*, *Erysipelotrichaceae*, *Sutterella*, *Barnesiellaceae*, *RF39*, *Veillonellaceae_Other*, *Roseburia*, *Acidaminococcus*, and *Paraprevotellaceae*.

### PICRUSt analysis

**Fig 4:**
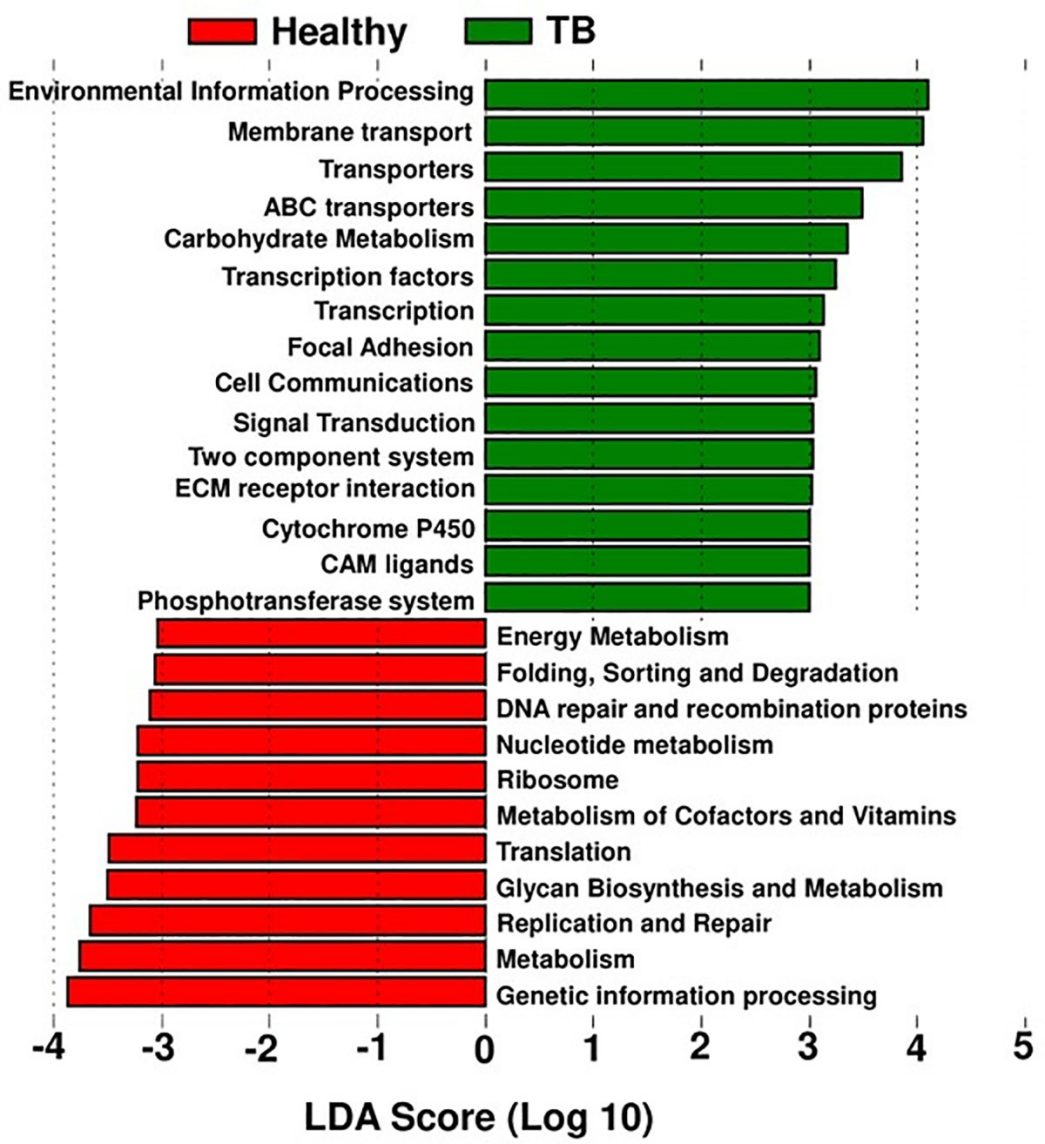
PICRUSt pathway prediction. The dominating pathways in TB patient group and healthy control group, are based on the dominant microbiota shown in Fig 3.

To study the potential function of gut microbiota, PICRUSt (Phylogenetic Investigation of Communities by Reconstruction of Unobserved States) were performed to predicate and identify differentially enriched pathways. The most notable pathways higher in TB patient group were environmental information processing, membrane transport, ABC transporters, adhesion, cell communication and signal transduction (Fig 4). Metabolic functions involved in the biosynthesis of nucleotides, glycan, cofactors and vitamin metabolism were depleted in TB patients compared to the healthy group (Fig 4).

The most notable pathways higher in TB patient group were environmental information processing, membrane transport, ABC transporters, adhesion, cell communication and signal transduction. Metabolic functions involved in the biosynthesis of nucleotides, glycan, cofactors and vitamin metabolism were depleted in TB patients compared to the healthy control group.

### Plasma antibodies against *M*.*tb*. correlate with significantly enriched bacteria in TB patients

**Fig 5:**
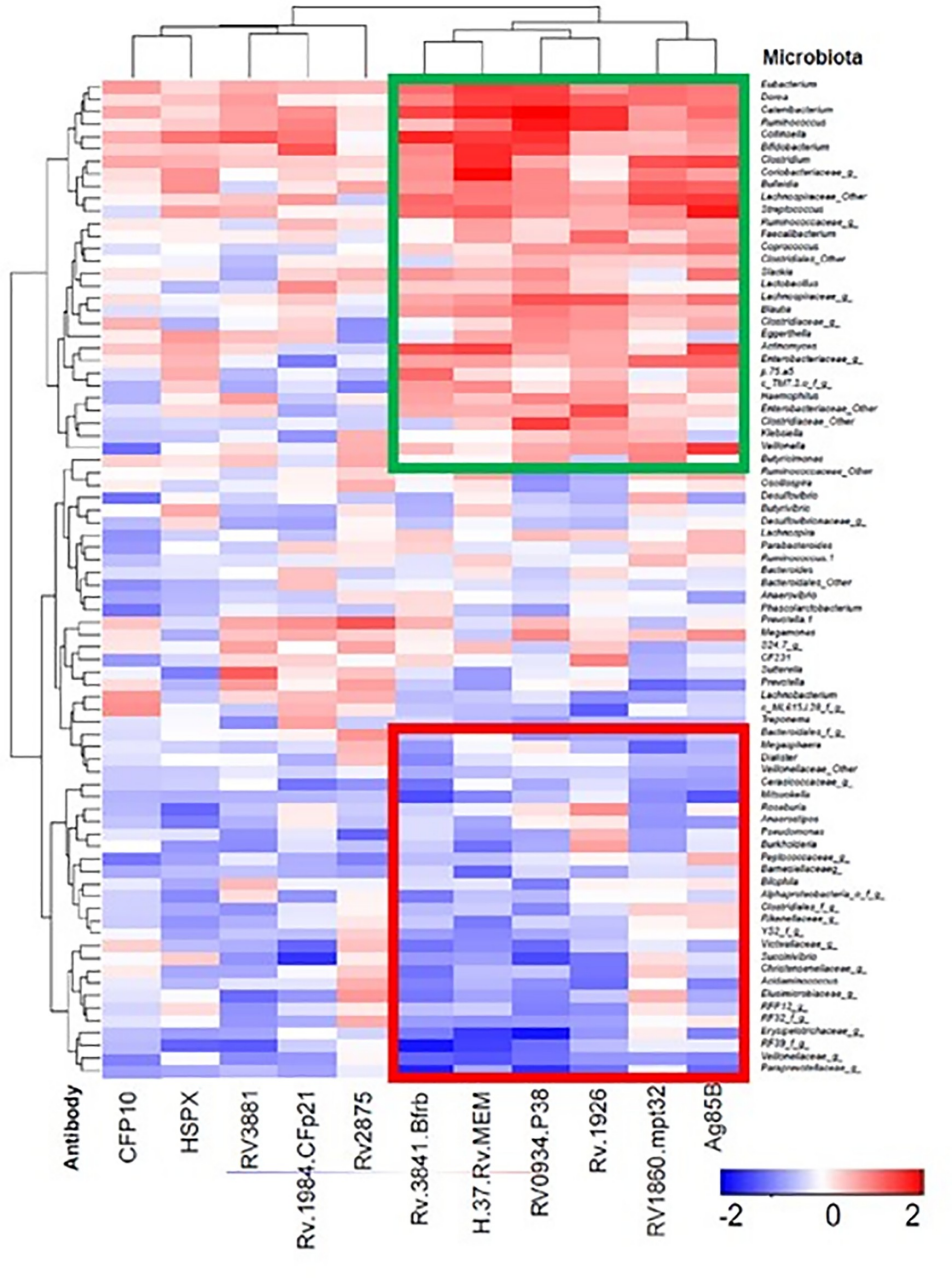
Correlations (Spearman) of anti-*M*. *tb*. antibodies in active-TB patients to gut microbiota. Plasma antibodies against 11 *M*. *tb*. antigens in plasma samples from active TB patients are shown on X-axis. Microbiota, at the genus, are shown on Y-axis. The Spearman’s ranked correlation test with false discovery rate (FDR) adjustment was used to test the microbiome-antibody correlation. Antibodies against *M*. *tb*. Antigens that showed positive correlation with the enriched bacterial genera are shown is red and those that displayed negative correlations with the genera are shown in blue. Statistical analysis was not performed for Fig 5 since it shows a general comparison of microbiota profiling between TB patients and healthy individuals. Microbiota most common in TB patients contain antibodies agaist antigens Rv0934, Rv1926c, Rv1860, Rv3841, and Rv1886c in addition to the whole membrane extract from H37Rv bacteria. The clusters that were boxed and specifically highlighted were identified visually based on the differences between healthy individuals and TB patients. Green box shows the microbiota profiles common in TB patients when the antibodies agaist 6 M.tb. antigens mentioned above are detected. Red box represents the microbiota profiling of healthy individuals who did not have antibodies against these antigens.

Multiplex analysis of plasma antibodies in active-TB patients was performed and their correlation with microbiota was evaluated using R programming with correlation test package of gplots and heatmap was generated (Fig 5). The clusters that were boxed and specifically highlighted were identified based on visual comparison between healthy individuals and TB patients. Antibodies against 5 *M*. *tb*. antigens (Rv0934, Rv1926c, Rv1860, Rv3841, and Rv1886c) in addition to the whole membrane extract from H37Rv bacteria (pathogenic laboratory strain), were highly correlated with several enriched bacterial genera in TB patients (Fig 5, green box). Antibodies against five other antigens displayed a lower level of correlation (Rv3874, Rv3881c, Rv2031c, Rv1984c, and Rv2875) (red box). Healthy individuals had no or low levels of antibodies and they correlate with the genera enriched in healthy individuals. The raw multiplex data of TB patients and healthy individuals is provided as supplementary data (S2 File).

### Copy number of butyryl CoA enzyme gene (*bcoA*)

**Fig 6:**
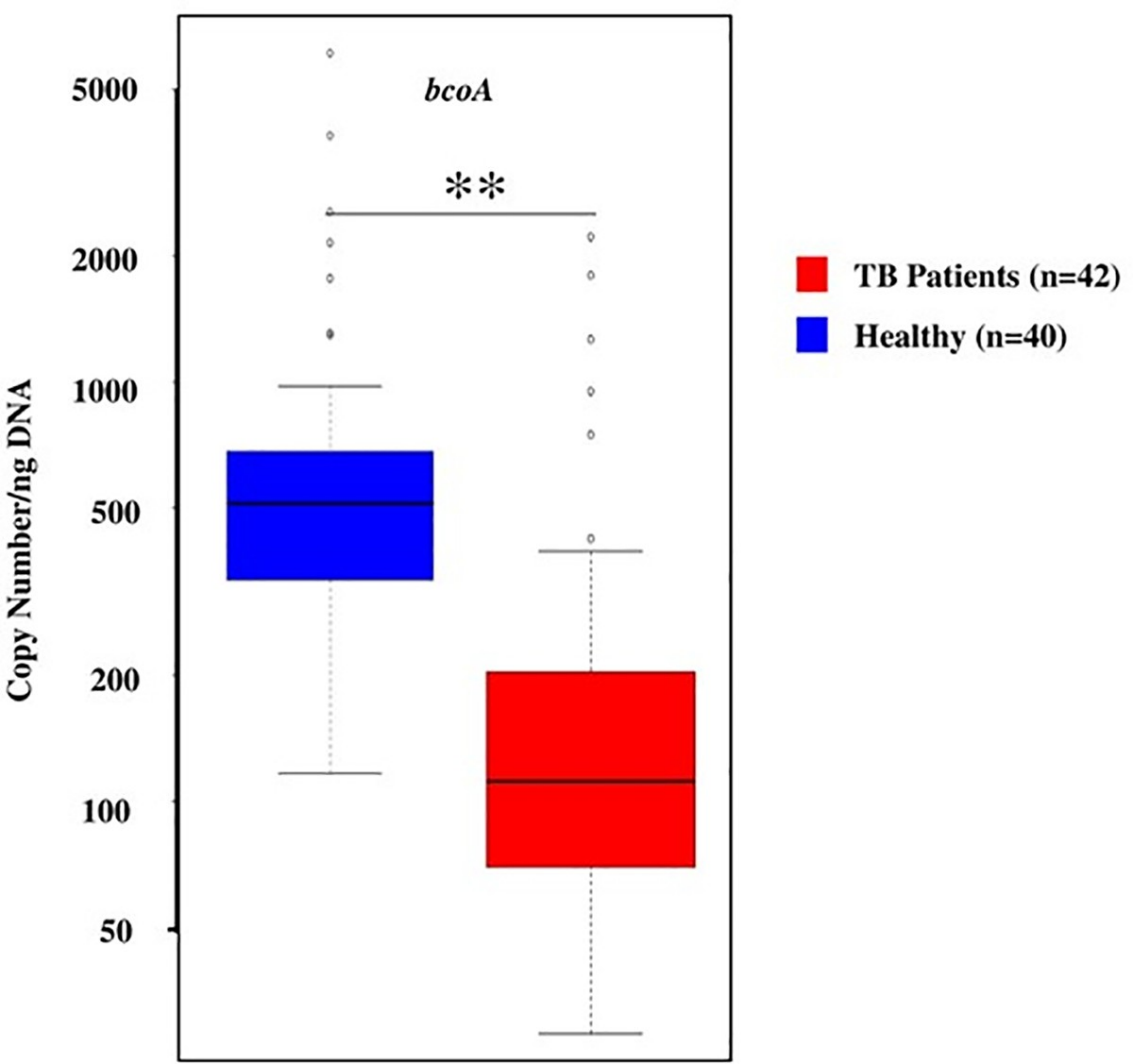
Copy number of *bcoA* gene in active-TB patients (red) and healthy individuals (blue), per ng of DNA. Data shown as a box and whisker plot. The line within the box represents the median value, the box represents the interquartile range (IQR), and bars represent the data spread. Copy number of *bcoA* gene was significantly (p-value: 0.004) lower in active-TB patients (Median: 111.8; IQR: 69.6–203.8) than in the healthy control group (Median: 515.05; IQR: 337.3–684.15); a five-fold reduction in gene copy number among the gut microbiota in TB patients.

The butyryl-coenzyme-A-CoA transferase (*bcoA*) gene was used for the quantification of butyrate producing microbiota [43]. Gene copy number for the enzyme *bcoA* enzyme is a marker for gut health (26, 27). Copy number of *bcoA* gene was significantly (p-value: 0.004) lower in active-TB patients (Median: 111.8; IQR: 69.6–203.8) than in the healthy control group (Median: 515.05; IQR: 337.3–684.15); a five-fold reduction in gene copy number among the gut microbiota in TB patients (Fig 6). This result indicates that under the conditions of active disease (TB), gut harbors five-fold lower levels of metabolites (e.g., butyrate) beneficial for normal health [34,35].

## Discussion

This study aims to define gut microbiota found in TB patients in comparison to healthy controls from the same indigenous area. TB is a complicated and chronic inflammatory disease with strong involvement of the host immune system. Infection with *M*. *tb*. is successfully contained in approximately 90% of the cases by the immune system resulting in latent infection, without the disease [3]. However, in 10% of the infected individuals' active disease occurs at some point, usually after a certain period of latency [3]. The trigger(s) that lead to the conversion of latent infection to active-TB are not well understood. The active disease has increasingly been viewed as an immune pathology underscoring the fact that the host immune system plays a major role in both latency and disease [3]. The gut microbiome is known to be involved in the immune system function [15,17,44]. The results of this study demonstrate that gut microbiota in TB patients are substantially altered in comparison to healthy controls, containing deleterious microbiome profiles. This dysbiosis in the host gut microbiome may influence the immune system as well as the disease outcome.

At the level of phyla, Fusobacteria, Actinobacteria, and Firmicutes were substantially enriched among TB patients (Fig 1); these phyla are known to contain many pathogenic bacterial species [28–32]. Whereas, two phyla, Bacteroidetes and Tenericutes, constituting a variety of beneficial commensal organisms, were diminished in the TB patients compared to the healthy controls, indicating their value in normal health [28–32]. In particular, the phylum Tenericutes, that contains many beneficial species, was substantially diminished in the TB patient group [45]. These results show that prevalence of beneficial microbiota is reduced in TB patients, in favor of those that are deleterious to health.

Samples from TB patients displayed differential clustering of gut microbiota compared to healthy controls, as analyzed by PCoA (Fig 2A). These results show that profiles of the gut-microbiota are similar within the active-TB patient group, but they are largely distinct from those in the healthy control group. In addition, PCoA plot showed (Fig 2A) greater diversity among microbial genera in TB patients compared to healthy controls.

Microbiome signatures associated with TB patient group in this study, are known to cause a variety of inflammatory diseases, malignancies, and adverse effects on enteric health (Fig 3). Examples are as follows:

**Families (**unknown genus within family contributed to significant difference in comparison with healthy controls): (a) *Ruminococcaceae*, microbial family enriched in TB patient group is associated with degradation of host mucus glycans. It is speculated that because of the loss of appetite in TB patients, overall food intake is low favoring enrichment of the members of this family that are capable of utilizing host mucus glycans, potentially compromising gut integrity and immunity [46]; (b) *Enterobacteriaceae* family is associated with inflammatory bowel disease (IBD) [47]; (c) *Erysipelotrichaceae* is linked to general pro-inflammatory milieu [48]. Enrichment of *Erysipelotrichaceae* has been reported in mouse model of active-TB [21,23]; (d) *Lachnospiraceae* family, like some of the other members of the phylum Firmicutes, is closely linked to obesity and gut inflammation in mouse model [49–52]. In direct relevance to TB, a recent study reported bacterial families *Lachnospiraceae* and *Clostridiaceae* are enriched in monkeys susceptible to experimental *M*.*tb* infection [53]—a member of *Clostridiaceae*, genus *Clostridium*, was enriched in TB patient group in this study (Fig 3); and (e) Members of *Coriobacteriaceae* have been associated with vaginosis, bacteremia, and periodontitis, and they are considered pathobionts—in the context of TB, the enrichment of this family of pathobionts may imply disease progression [54].

**Genera** (a) The genera, *Faecalibacterium* and *Eubacterium* were enriched in the TB patient group. These findings are corroborated by earlier reports in the Indian sub-continenet TB patient population [46]; (b) *Catenibacterium*, is reported to be enriched in HIV-infected individuals [55]; (c) *Bifidobacterium* was enriched in the TB patient group in this study, a known opportunistic pathogen that causes bacteremia in immunocompromised patients, or those with a compromised intestinal barrier [56]. Other members of the family, *Bifidobacteria*, are known to carry genes capable of conferring antibiotic-resistance to anti-tubercular drugs [57]; (d) *Eggerthella* is an anaerobic gram-positive bacillus associated with polymicrobial intraabdominal infections [58]; (e) *Catenibacterium*, (f) *Collinsella*, and (g) *Eggerthella*, were found to be enriched in the gut microbiota of TB patients in this study, they are reported to be associated with rheumatoid arthritis, another inflammatory disease [59,60]; and (h) *Burkholderia* is associated with pneumonia-derived melioidosis [61].

The enrichment of above microbiota in TB patients, known to be associated with a variety of inflammatory and immune dysfunction diseases, suggests they may contribute to TB pathogenesis. We speculate on the basis of our exploratory findings in this study they may also contribute to TB-related morbidity, general health deterioration, and weight loss but this needs to be investigated in a separate study.

The gut microbiota enriched in healthy controls are as follows: (a) Genus *Prevotella* (phylum *Bacteroidetes*, Fig 1); dominance of *Prevotella* in our study is similar to the prior characterization of gut microbiome composition reported in healthy individuals in the Indian sub-continent populations [46,62–64]; (b) Genus *Roseburia* was also enriched in the healthy control group—previously reported to be prevalent among the gut microbiome of healthy population in the Indian sub-continent [62]; (c) *Mitsuokella*, and (d) *Succinivibrio*, are instrumental in fermentation of fiber and polysaccharides from vegetables, contributing to normal human health [65–67].

A previously published study about the changes in the gut microbiome of Chinese cohort presented similar findings with an increase of the phylum *Actinobacteria* and decrease of phylum *Bacteroidetes* in TB patients. Interestingly, the phylum *Firmicutes* which was substantially enriched among TB patients in the Pakistani cohort was found to be significantly reduced in the Chinese cohort [74]. In a separate study, Hu and Colleagues found no significant difference in the relative abundance of the members of the phyla *Bacteroidetes* and *Firmicutes* between the Tuberculosis group and the healthy controls [19–23]. The enrichment of *Faecalibacterium* and *Eubacterium* in TB patients in our study is similar to the findings stated in another independent study involving an Indian cohort. However, we found an abundance of *Bifidobacterium* in the TB patients whereas their enrichment was observed in the healthy controls of the Indian cohort [46,62,63]. Additionally, high-throughput 16S rRNA gene sequencing data from a 2016 study revealed a significantly higher relative abundance of *Firmicutes* and *Actinobacteria* in the TB samples of Indian origin [68]. 16S rRNA Pyrosequencing analysis of Sputum samples disclosed a different microbial profile in Chinese patients where *Proteobacteria* and *Bacteroidetes* were reported to be more enriched in the TB samples and *Firmicutes* being abundant in the controls [69]. Based on the determination of the dominant microbiota in this study (Fig 3), we investigated potential of their contribution to metabolic pathways in the TB patient group (Fig 4). Not surprisingly, the most notable pathways dominating in TB patients (e.g., environmental information processing, membrane transport, ABC transporters, transcription, signal transduction, etc.) have been reported to be upregulated in other diseases e.g., auto inflammatory and autoimmune disorders, chronic periodontitis, cystic fibrosis, Alzheimer’s disease, etc. [70–73]. Cytochrome P450 pathway is known to have been upregulated in several cancers, diabetes, atherosclerosis [71]. In contrast, metabolic functions observed in the healthy control group, involved in the biosynthesis of nucleotides, glycan and cofactors and vitamin metabolism, were depleted in TB patients [46].

The potential for adverse effects on gut health, by the gut microbiota found in TB patients, is also supported by the data on *bcoA* gene copy number (*bcoA* supports production of metabolite, butyrate, known to be important in gut health) in the fecal DNA (Fig 6). The gene copy number in active-TB patients was 111.8 (Median) ranging from 69.6–203.8 (IQR), while in healthy individuals it was 515.05 (Median) ranging from 337.3–684.15 (IQR). This five-fold reduction in gene copy number among the gut microbiota in active-TB patients suggests that reduction in butyrate production, a key metabolite involved in maintaining proper gut health, puts active-TB patients at a disadvantage concerning the integrity of the digestive tract. Butyrate is a four-carbon microbial fermentation product that serves as a preferential energy source for the gut epithelial cells [74]. The severity of inflammatory and metabolic diseases is in general inversely proportional to butyrate production [75–77]. It is possible that certain butyrate producing bacteria such as Faecalibacterium and Eubacterium abundance were increased in TB patients. However, it needs further investigation or high throughput metagenomic sequencing to identify the selective butyrate producing bacteria present in healthy controls and/or TB patients.

In addition, we examined the correlation of plasma antibodies, against eleven *M*. *tb*. antigens, that collectively serve as excellent blood-based biomarkers for active-TB [18,26,78]. The correlative pattern of antibodies with the microbiome profiles reflected that of the one observed between active-TB patients and healthy individuals. That is, the antibodies showed a correlation with the same microbial genera that were prominently seen dominating in the active-TB patients (Fig 5). This correlation suggests that in addition to the plasma antibodies, or in combination with, gut microbiota may be used as potential active-TB biomarkers. Comorbidity may have an influence on this signature when statistically confounding factors such as diabetes have been taken into account. To our knowledge, this is the first documentation of possible associations of the gut microbiota with blood-immune biomarkers in the *M*.*tb*. infection. This study presents an analysis of active-TB related microbiome profiles in a TB endemic country, Pakistan.

This study demonstrates dysbiosis of the gut microbiome in active-TB patients. As only TB patients and healthy controls were included in this study, it is not clear if the gut microbial profiles found in TB patients would be distinct from other non-TB pulmonary disease patients e.g., pneumonia, COPD etc. Our future studies will include these disease groups. In addition, we will perform deeper microbiome analysis at the species level. Metaproteomics profiling will also be performed to depict the functional features of the gut microbiome in more detail in addition to the metagenomics profiling presented in this paper.

## Supporting information

S1 File Clinical and demographic data of TB patients and healthy individuals. (XLSX)

S2 File Multiplex data of antibodies for TB patients and healthy individuals. (XLSX)

S3 File BcoA data of TB patients and healthy individuals. (XLSX)

S4 File Relative abundance data fir 16S rRNA sequencing of TB patients and healthy individuals. (XLSX)

S5 File List of genera enriched in TB patients and healthy individuals shown in Fig 5. (XLSX)
